# Managing Cross-Border Conflicts Through Volunteer Commitment: A Comparative Study Between Religious and Non-profit Organizations in the San Diego–Tijuana Area

**DOI:** 10.3389/fpsyg.2019.02978

**Published:** 2020-01-28

**Authors:** Luis Manuel Cerdá Suárez, Jesús Alberto Valero Matas, Martha Cecilia Jaramillo Cardona, Margarita Ramírez Ramirez

**Affiliations:** ^1^Faculty of Business and Communication, International University of La Rioja, Logroño, Spain; ^2^Department of Sociology and Social Work, University of Valladolid, Palencia, Spain; ^3^Faculty of Economics and International Relations, Autonomous University of Baja California, Tijuana, Mexico; ^4^Faculty of Accounting and Administration, Autonomous University of Baja California, Tijuana, Mexico

**Keywords:** immigration, cross-border conflict, non-profit organizations, religious organizations, diversity management, metropolitan area, volunteer commitment

## Abstract

San Diego and Tijuana configure two big cities that have faced each other across the international boundary between United States and Mexico for over 180 years. Within this context, the relationship emerging at the border can be characterized under different categories of individual, social, economic, and political situations connecting each side. Additionally, in recent years, the literature on cross-border conflicts has extensively focused on volunteers as informal agents helping children and other groups of population, but relatively little research has addressed the practical and managerial work and implications of the volunteers themselves. As actors of cross-border communities, volunteers play a relevant role in effective short-term migrants’ settlement, but it is also observed that the profile of volunteers in religious organizations differs from those belonging to non-profit institutions. Grounded on the theories of Planned Behavior and Reasoned Action suggesting that intentions to cooperate with non-government institutions are influenced directly by attitudinal values and indirectly by their beliefs related to social conflicts, this paper analyzes the nature of volunteer commitment in religious and non-profit organizations (NPOs) providing information about managerial practices for newly arrived migrants. The main purpose of this study is to investigate the relevance of volunteer commitment as an instrument for managing cross-border conflicts in the particular context of San Diego and Tijuana Area. Based on research using interview data collected from beneficiaries by volunteers, institutional representatives, and documentary references, this manuscript highlights a psychological and individual-centric perspective of volunteer commitment, but it also explores a collective communicative action expanding the range of relevant voices in decisions about volunteering. Moreover, this study provides new insights into how organizational and relational elements impact sustainable volunteer management and points out the role played by attitudes toward non-government institutions such as religious and NPOs demonstrating the relevance of volunteer commitment, transforming part of the positive attitude toward social problems into a significant intention to cooperation. Understanding the importance of the organization’s images in order to attract volunteers, these results show that commitment may become a key determinant of the volunteers’ identity linked to strategies devoted to organizational activities.

## Introduction

In the last two decades, the proliferation of cross-border conflicts and the relevance of multilevel humanitarian actions have witnessed a great growth around the world (Bastida et al., 2008; González, 2014; Abdul, 2015; Gustavo, 2015; Suh et al., 2017; Silva, 2019). These phenomena represent more than just important repercussions in the manner in which the mass media, managers, and directors in non-profit organizations (NPOs) put volunteering into context and are open to interpretation focused on the availability of evidences on political, economic, and social effects of migrants (Taylor, 2007; Metz et al., 2017; McAllum, 2018; Hövermann and Messner, 2019; Lu et al., 2019).

From a comprehensive perspective on management in NPOs, the Organizational Behavior discipline became compulsory, exploring the relevance of Human Resources (HR) practices on intentions, attitudes, and behavioral outcomes of volunteers. Several practitioners and scholars have evidenced different questions and insights into how NPOs can promote positive and sustainable attitudes, intentions to collaborate, and behaviors on volunteers and, as a consequence, have an advantage in their development and long-term retention (Sparrow, 2001; Alatrista and Arrowsmith, 2004; Ariza-Montes et al., 2015; Rhodes et al., 2015; Şlusarciuc, 2015; Alfes et al., 2017; Bartram et al., 2017). However, conceptualizations and measurements of some terms are very difficult to reach in that discipline. For this reason, developing conceptual, theoretical, and empirical frameworks oriented to commitment, it is very relevant as a comprehensive starting point regarding the existing Business Management literature.

There is an important focus on the operational side perspective in terms of the “managerial decisions” on the role played by leaders and directors in NPOs. Managers acting as gatekeepers who are in charge of recruitment, retention, and continuous motivation of HR in their organizations are becoming critical for their success and survival. Moreover, they have to take into account carefully what managerial changes need to be done in a calculated manner to raise the organizational performance, volunteer satisfaction, and obviously its loyalty (Bendapudi et al., 1996; Ridder and McCandless, 2010; Brudney and Meijs, 2014; Ariza-Montes et al., 2018).

However, little investigation has been done to date related to managerial decisions in order to be more responsible to retention of staffs across voluntary organizations (Hernández, 2008; Ohana and Meyer, 2016; Shandra, 2017). These managers’ opinions are not only based on “attitudinal” factors, but rather on “normative” attributes related to feelings and sentiments of obligation and gratitude, which have come to be linked to activities that promote long-lasting behaviors (Wymer and Starnes, 2001; Spruyt et al., 2018; Lindley, 2019). Nowadays, the highly turbulent social context forces managers in NPOs to facilitate the volunteer commitment and identify the aspects that impulse the retention of NPO employees (McCormick and Donohue, 2016). Managerial decisions in NPOs try to be quite structured in order to implement actions and policies based on a formalized, systematic, and strategic approach implementing techniques to positively contribute to volunteer satisfaction and build sustainable communities among management practitioners, governments and communities across different contexts (Ariza-Montes et al., 2015; Metz et al., 2017; Watts, 2018; Lindley, 2019).

As most theoretical and empirical studies in NPOs are related to the nature of practices undertaken and links between volunteers and NPOs, this paper deals with an original topic describing volunteer commitment and behavior with practices enhancing volunteers’ performance in the behaviors they act, their role, and the effectiveness of the NPOs in managing cross-border conflicts (Wymer and Starnes, 2001; Abdul, 2015; Metz et al., 2017). The purpose of this manuscript is to extend comprehensive frameworks, drawing particularly on terms linked to the literature on organizational behavior and management practices pertaining to NPOs. The conceptualization that we have described in this research is related to disciplines such as non-profit and HR management, sociology, psychology, and organizational behavior, but draws particularly on the literature of the theory of planned behavior and reasoned action (Ajzen and Fishbein, 1980; Alves et al., 2015; Bloemraad and Terriquez, 2016). Additionally, we present a conceptualization of commitment grounded on Becker’s (1960) theory, cognitive dissonance theory of Festinger (1957), and the social exchange theory due to the relevance of political demands oriented to ensure a minimum level of civil rights for a social group and consequently the role these actions play in transforming individual’s opinions.

The main challenge for NPOs is on human or community development, but Social and Nonprofit Marketing and Organizational Behavior emerged for similar reasons (Zarzuela and Antón, 2015). Both disciplines are frequently in search of actions for effective and relevant changes in organizations and institutions including NPOs. In this work, a wide temporal representation of cross-spatial nature was conducted at NPOs in the San Diego–Tijuana Area, within the framework of an international research project focused in health NPOs. The final aim of this research is oriented to assess the influence of different valued combinations of attitudinal and normative-focused attributes on implications of volunteer commitment and behavior, considering some particularities of cross-border areas.

This research is rooted in a behavioral framework based on theories described by Ajzen and Fishbein (1980). According to the theoretical approach of Planned Behavior, Fishbein and Ajzen (1975) and Ajzen and Fishbein (1980) pointed out that intentions affect behaviors. MacMillan et al. (2005) and Lee et al. (2014) point out that two main terms of this theoretical framework – perceived behavioral control and attitudes – influence behaviors. This manuscript is particularly guided by attitudinal and normative values based on the social exchange theory that include several elements such as gratitude and moral obligation (Miles and Vaisey, 2015; Whitehead and Stroope, 2015). In terms of practical implications, this manuscript is focused on empirical evidences demonstrating how NPOs can promote intentions and sustainable behaviors on volunteers facilitating the managerial processes through which HR practices exert their influence (Sheppard et al., 1988; Stirling et al., 2011; Miles and Vaisey, 2015; Rhodes et al., 2015).

The outline of this manuscript is as follows. First, a review of the literature on topics, conceptual framework and research agenda is conducted. Second, a methodological design and procedure of research and results are described. Third, an additional section is devoted to discussion, and finally, contributions, conclusions, implications, limitations, and further research are presented in these pages.

## Literature Review and Hypotheses

Several authors point out that volunteering is a relevant behavior configured by four features distinguishing it from other social actions (Allen and Meyer, 1990, 1996; Van Vuuren et al., 2008): (1) it is a planned behavior; (2) it is a long-term behavior; (3) it is a non-obligatory helping behavior; and (4) it takes place within an institution. According to Stazyk et al. (2011), theories of Planned Behavior and Reasoned Action’s theoretical and conceptual framework are typically selected when investigating volunteer commitment, satisfaction, intentions to stay, and behaviors, mostly among employees and volunteers but also in management contexts. In particular, Theory of Planned Behavior (Fishbein and Ajzen, 1975; Ajzen and Fishbein, 1980; Burnkrant and Page, 1982) has been applied frequently in volunteering research to analyze intentions to stay among volunteers of NPOs, particular attitudes reflecting a person’s belief about a behavior (Ariza-Montes et al., 2015; Hansen and Kjeldsen, 2017), volunteer retention and loyalty (Yavas and Riecken, 1997), intentions to stay (Ranganathan and Henley, 2008; Van Vuuren et al., 2008), subjective norms reflecting beliefs of others in terms of particular behaviors (Cohen, 2011), perceived behavioral control as a determinant variable of the intention to cooperate with the organization (Kim and Lee, 2007; Ranganathan and Henley, 2008), and person’s past experience, opportunities, resources, and barriers to performing behaviors (Lee et al., 2014; Ariza-Montes et al., 2018).

While this theoretical framework has been applied in business and management contexts, such studies are scarce in the cross-border literature. The Theory of Planned Behavior is based on the hypothesis that human beings act rationally, make systematic and frequent use of information, and consider the further implications of their actions before engaging in a specific behavior; that is, the stronger a person’s intention to perform a behavior, the more likely that person will perform a particular behavior. For instance, some of the specific management contexts of application related to this conceptual framework in NPOs consider the following: nurses’ intentions and behaviors to care (Van Vuuren et al., 2008); preferences for pollution reduction of managers (Amos, 1982; Alatrista and Arrowsmith, 2004); younger ones’ intentions and behaviors (Briggs et al., 2007; Zarzuela and Antón, 2015; Chordiya et al., 2017); unethical and fraudulent financial behavior (Van Vuuren et al., 2008; Gupta, 2017); sports events (Lee et al., 2014); knowledge-sharing perceptions, behaviors, and actions of managers (Kim and Lee, 2007); and employee decisions about using new technologies (MacMillan et al., 2005).

Additionally, drawing on the Theory of Reasoned Action (Ajzen and Fishbein, 1980), several authors point out that an awareness of social conflicts, attitudes toward such problems, and NPOs’ environment interact to characterize the process of social commitment (Zarzuela and Antón, 2015). From this theoretical perspective, a greater awareness of social conflicts and problems causes a positive attitude toward them and consequently it will engender a greater likelihood of volunteers in terms of a social commitment.

Despite the extensive research, interest in this topic remains because of individuals and volunteers simply do not respond to new marketing actions and strategies in the same way when it comes to NPOs. Moreover, it appears that no studies have used this approach to investigate volunteers’ commitments and behaviors in the context of cross-border conflicts. For this research, the conceptual framework, here, exposed seems more relevant for situations where individuals do not perceive themselves as having absolute control over their behaviors (Ramlee et al., 2016), as might occur in volunteering and volunteers’ intentions as a specific form of helping activities in political and social conflicts (Sparrow, 2001; McFarland and Thomas, 2006; Henderson and Sowa, 2019; Instituto Nacional de Migración [INM], 2019; Silva, 2019).

### Attitudinal Commitment and Volunteer Behavior

In Business Management and Social Marketing studies, the relevance of the volunteer commitment has been evidenced by several researchers as an important indicator for success in the NPOs. In the specific context of social marketing strategies, a NGO represents “the particular ‘citizens’ way of expressing their perceptions by forming groups which seek altruistic, social and caring goals, benefiting those who are, excluded, underprivileged, and marginalized from society.” Similarly, volunteer commitment is defined as “a link with the organization that involves either behavior or attitude” (Beerli et al., 2004; Dawley et al., 2005; Amos and Weathington, 2008). For instance, González and Guillén (2008) and Grant et al. (2008) evaluated several dimensions and attributes that compose the topic of volunteer commitment and motivate the volunteer satisfaction and its behavior.

Relevant aspects such as individual’s identification with the value system of the organization and internalization of attitudes increasingly became challenges for volunteers and NPOs. In this particular case, the concept of internalization involves more than loyalty, it implies a proactive relationship with the institution as a manner to serve one’s own interests. This is the justification why managers have to communicate available information in NPOs and their websites in order to develop a plan for recruiting or involving volunteers (Jaros et al., 1993; Harrison et al., 2017; Jaros, 2017).

In Organizational Behavior literature, commitment is characterized as an emotional (that is, affective), ethical, and rational phenomenon (Boezeman and Ellemers, 2008; Alfes et al., 2017). Considering the different interpretations, the nature of the bonds, and the main differences among contexts of analysis, all the authors agree that this term is well defined as a “link between the organization and the individual that involves either attitude or behavior” (Juaneda et al., 2017). Consequently, volunteer commitment is conceptualized as a range of perspectives from the emotional to the instrumental dimension, reflecting different components of and therefore scales of measurement (Malhotra, 2005; Jaros, 2017; Juaneda et al., 2017). Moreover, commitment is an emotional and affective response of the individual as a consequence of a global evaluation of all the attributes that determine the volunteer helping behavior; that is, commitment is a signal of the individual favorable affection to the ONG (Galindo-Kuhn and Guzley, 2002). From an extensive perspective, committed volunteers and staffs in NPOs can stay longer interacting with users through the positive experiences or generating particular opinions to the others.

Although in the field of management of volunteers, the best HR practices do not consider volunteer satisfaction and its commitment, several authors point out that both constructs have a relevant effect on behavior (Ariza-Montes et al., 2015; McCormick and Donohue, 2016). That is, different attitudinal aspects (identification and internalization, for example: Juaneda et al., 2017) are frequently used to increase the volunteer commitment in NPOs, and as a consequence, they cause high levels of satisfaction, intentions to stay, and participation. For instance, Ajzen and Fishbein (1980) and Malhotra (2005) characterized attitudinal attributes of volunteer commitment showing the relevance of constructs such as social norms, intentions, and motivations. Furthermore, identification and internalization can generate positive feelings when the volunteer wants to remain in the organization; this is especially relevant given the socio-emotional need, motivation, favorable experiences, and voluntary contributions in terms of time, expertise, and energy (Ohana and Meyer, 2016; Ariza-Montes et al., 2018). Available research and the literature review on this topic show that the organization’s affective bonds represent a higher level of commitment in a particular context of scarce resources and can solve the problem faced by managers in NPOs of how to motivate employees committed without offering them the highest salaries (Ohana and Meyer, 2016). Additionally, perceived emotional benefits can generate higher levels of involvement, engagement and helping behaviors for employees and volunteers (Meyer et al., 2004; Valéau et al., 2013; Benevene et al., 2018).

To sum up, in this first characterization of commitment, attitudinal values are perceived as impacting volunteer helping behavior. When serving a cause or specific social mission satisfies the expectations of volunteers and employees, their helping behaviors is likely to increase (Briggs et al., 2007; Vecina et al., 2012). The review of the literature on commitment carried out in the context of this work allows one to analyze the most relevant attributes impacting on the volunteer helping behavior in NPOs in the workplace, in order to promote and capitalize affective commitment as input to volunteer behavior. Several specific hypotheses were developed for exploring the issue discussed previously.

Both the theory of Reasoned Action and seminal literature in volunteering suggest the need to differentiate between attitude toward behavior and also attitude toward organizations. According to several authors, attitude toward social problems become more general than attitude toward NGOs (Briggs et al., 2007; Wilson, 2012), highlighting that both types of attitude are relevant indicators of behavior as evidenced by research in nonprofit and social marketing. Moreover, NPOs can create a positive image to get individuals’ social commitment and helping behavior (Meyer and Allen, 1984, 1991; Mayer and Schoorman, 1992; Meyer et al., 1993; Briggs et al., 2007; Wilson, 2012).

### Normative Commitment and Volunteer Behavior

In the context of NPOs (including permanent programs and crisis actions), gratitude and moral obligation evidenced by volunteers are evidenced as two relevant factors in the information search process for recruitment and volunteers’ intention to stay and cooperate with the organization (Porter et al., 1974; Pearce, 1993; Briggs et al., 2007). In addition, for managers in NPOs, these normative judgments reinforce the behaviors of those who follow these moral principles, rules and norms (Malhotra, 2005; Juaneda et al., 2017).

Regarding the normative features involved in volunteer commitment and helping behavior, several authors point out that, when volunteers are committed, they feel compelled to stay connected with the institution. From this conceptual approach, different attributes such as gratitude and moral obligation characterizing commitment can promote its preference; as a result, these moral conceptualizations are relevant in NPOs considering the individuals’ opinions about political demands and social pressure oriented to ensure a minimum level of rights for a particular group or society in general. Thus, pertaining to one of these NPOs is a public signal of certain convictions (Neuberg et al., 1997; Alatrista and Arrowsmith, 2004). According to Becker (1960) and Blau (1964), social exchange theory is of significant relevance: a number of studies have defined gratitude as a sense of obligation to NPO due to the feelings of receiving more than what has been given by the individual.

Additionally, according to the cognitive dissonance theory of Festinger (1957), the consideration of moral obligation related to volunteer commitment identify the tendency of individuals committed to participate on a social project in order to avoid contradictions on his/her line of action that was already begun. That is, in terms of this desire for a great internal consistency in volunteers, beliefs and attitudes of individuals may not only impact on their behaviors, but also they are a result of it. This proposition defends that attitudes are private and relatively malleable. By contrast, behaviors are more irrevocable and public (Ohana and Meyer, 2016; Juaneda et al., 2017).

The final outcome of volunteer commitment is in behavioral terms, meaning that volunteers link to a particular NPO in the expectation that they will contribute to find positive experiences in collective contexts and achieve organizational goals and where social ties and moral obligations become relevant (Miles and Upenieks, 2018). Thus, it is supposed that these normative aspects will have a positive impact on the volunteer helping behavior when NPOs are managing humanitarian actions and social conflicts.

As well as in the Social Marketing discipline, there is no doubt that without donors and volunteers devoting effort, time, and funds, NPOs could not survive: in the social exchange theory, it is necessary to assess the normative values in order to considerer its relevance on volunteer helping behavior in the development of their promotional and humanitarian tasks (Baron and Kenny, 1986; Reed and Selbee, 2000; Reed et al., 2007; Miles and Upenieks, 2018). Therefore, it seems reasonable to consider that a high level of “normative bonds,” that is, the greater importance of the gratitude and moral obligation aspects in order to motivate their feelings, the higher level of volunteer commitment in their helping behaviors. From this perspective, according to Jaros (2017) and Juaneda et al. (2017), several normative features such as gratitude, individual’s sense of moral obligation toward the NPO, and obviously perceived social problems reveal higher scores on commitment influencing volunteer helping behaviors.

In research on Social Marketing and Organizational Behavior, only few authors have conducted investigation related to the relationship between these conceptualizations. Taking into account the high level of available information shown by the perceived positive features of commitment to capture the interest of volunteers, the abovementioned literature review allows us to consider our research proposal based on features related to achieve a given volunteer helping behavior.

### Research Proposal and Hypotheses

According to several investigations in which the relationship among attitudinal and normative attributes related to commitment, intention to stay in the organization, and volunteer helping behavior in NPOs is identified (e.g., Valéau et al., 2013; Juaneda et al., 2017), the review of the literature allows us to establish two substantive hypotheses, HA and HB respectively:

*HA*: The individual’s attitudinal attributes configuring commitment toward NPOs will lead to a higher volunteer helping behavior.
*HB*: The individual’s normative attributes configuring commitment toward NPOs will lead to a higher volunteer helping behavior.

In order to operationalize our research proposal derived from this integrative theoretical framework, we present the following auxiliary hypotheses related to HA and HB:

*H1*: The individual’s identification with the value system of the organization will have a direct effect on the volunteer helping behavior.
*H2*: The individual’s internalization of the value system of the organization will have a direct effect on the volunteer helping behavior.
*H3*: The individual’s attitude toward NPOs will have a direct effect on the volunteer helping behavior.
*H4*: The individual’s sense of gratitude toward the NPO will have a direct effect on the volunteer helping behavior.
*H5*: The individual’s sense of moral obligation toward the NPO will have a direct effect on the volunteer helping behavior.
*H6*: The individual’s attitude toward social problems will have a direct effect on the volunteer helping behavior.

## Materials and Methods

In order to evaluate the evidence of the relationship between volunteer commitment and helping behavior, this research was applied on beneficiaries in religious and civil NPOs that, for a long time, facilitate health located in the San Diego–Tijuana area. According to Metz et al. (2017), it becomes relevant to know why it is important to beneficiaries. In contrast to the economic value of volunteering to the volunteers and communities, to date, little is known about theses value to migrants and beneficiaries.

Two specific aspects of the methodological process should be mentioned in this research regarding:

1. the participants, and
2. the instrument, detailed procedure and scope of this research.

### Participants

Regarding the participants in this research, in order to study the proposal between the concepts, here, described, this investigation was tested in migrant shelters located in the San Diego–Tijuana area, named Otay Mesa and San Ysidro Port of Entry.

Our research was applied in the following religious institutions: the Centro Madre Assunta and Casa de los Migrantes. Both religious institutions were the first in Tijuana to establish a method of providing dignified assistance to children and women who are migrating from Central and Southern Mexico or have been displaced after deportation from the United States. As civil institutions, YMCA and Módulo Fronterizo Tijuana were considered for this research. In particular, this research was carried out in migrant shelters in Tijuana run by civil society organizations and institutions such as Protestant and Catholic churches (INEGI, 2017).

According to the comparative design of this research between religious and civil organizations, the total number of participants was 1535, proportionally stratified by type of NPO as a sampling procedure (random quota sampling); migrants were asked to evaluate the volunteer commitment and helping behavior in the shelters. In order to collect a diverse data set, the participants were contacted from a range of shelters that offered volunteering experiences and situations at different locations, as a form of triangulation to increase validity. This comparative design includes similarities and differences, which can provide a conceptual basis as well as a practical implication at each NPO in particular.

The participants were contacted from a census in each shelter; in order to appreciate the importance of the volunteer commitment, a simple random sampling method was used as a selection procedure for the sampling units. According to the literature on this topic (Bentler and Bonett, 1980; Wisner et al., 2005; Valéau et al., 2013; Doucet and Lee, 2015; Tekingündüz et al., 2017; Engel, 2018), previous managers and volunteers with experience and knowledge in NPOs were contacted in order to evaluate if our instrument was adequate to these objectives (that is, face validity).

The instrument (questionnaire) was pre-tested in a sample of convenience among beneficiaries with similar characteristics to the final target, on which the questionnaire was applied. The particular scale on volunteer commitment was derived from the aforementioned literature (Schlegelmilch and Tynan, 1989; Malhotra, 2005; Sargeant et al., 2006; Doucet and Lee, 2015; Metz et al., 2017).

### Instrument, Data Collection, and Scope of Study

Regarding the detailed sources of information and the procedure for this research, respondents were asked to fill in a questionnaire (data collection procedure) with the following information:

Part I: “Volunteer helping behavior,”
Part II: “Attitudinal and normative-focused commitment,” and
Part III: “Identification data of the respondent.”

In this research, the main variables (Parts I and II) were collected in the questionnaire with a Likert-type scale ranging from 1 (strongly disagree) to 10 (strongly agree).

Items in the scale on volunteer commitment were defined as a structured questionnaire of closed questions, with support in the results of the qualitative methodology applying interviews in a preliminary test, and derived from the particular context where it was the fieldwork.

According to the aforementioned literature on volunteer commitment, we used a reduced adaptation of the Juaneda et al. (2017) scale in terms of main dimensions for three reasons: (1) it resulted in a highly operative measure including only a very small number of attitudinal and normative features; (2) it presented appropriate psychometric properties from the literature point of view; and (3) the scope of application was very similar (religious and civil institutions as NPOs). Additionally, we included attitude toward NPOs and social problems according to Zarzuela and Antón (2015) scale. This adapted scale considered volunteer commitment composing the characteristics reported in the previous literature, i.e., attitudinal (individual’s identification and internalization of the value system of the organization, attitude toward NPOs) and normative features (gratitude, moral obligations toward the NPO, and individual’s attitude toward social problems: see details in the Supplementary Material).

This research used Briggs et al.’s (2007) approach to analyze the impact of commitment in NPOs on the two relationships proposed in this manuscript: (1) between attitudinal values and volunteer helping behavior, and (2) between normative values and volunteer helping behavior. In addition, a criterion of simplicity was used to consider responses on commitment into categories (a high or low two-level for perceptions) based on our descriptive analysis.

Related to the data collection procedure, the longitudinal fieldwork was conducted in the San Diego–Tijuana area by Doctors of the World–Médecins du Monde (MdM), a movement of activists who provide care, bear witness, and support social change in Tijuana from 2006 through diverse medical programs and evidence-based initiatives, enabling migrants to access health.

In 2017, Mexican authorities declared a “humanitarian crisis” for several reasons in response to the large number of migrants in Tijuana, also known as the Central American migrant caravans. Previously, Amnesty International reported that between 2007 and 2012, several Central American countries had the highest average homicide rates in the world and Pueblo Sin Fronteras noted that such caravans began arriving several years earlier (Silva, 2019).

Tijuana is recognized as the most important border city in Mexico and a complex dynamics have shaped it not only in a demographic sense but also in social, economic, and cultural terms (Mendoza, 2017; Silva, 2019). In 2015, this city was the third most densely populated municipality in Mexico (INEGI, 2017). Since 2016, the migratory pattern evidences Haitians, Mexican displaced and detained by U.S. Immigration and Customs Enforcement (ICE) while crossing the border, and Central Americans arriving by caravans.

Table 1 shows the specific methodological procedure by documentary references and interviews for designing the questionnaire used in this research. Additionally, a quantitative methodology was applied to collect the final data.

**TABLE 1:** Methodology of this research.

**Analysis**	**Methods**	**Techniques**
• Documentary, face validity	• Literature review, qualitative research	• Bibliographic analysis, in-depth interviews
• Reliability, construct validity	• Quantitative research (analysis of overall reliability and initial factor validity)	• Descriptive statistics • Cronbach’s alpha • Item-total correlation • Factor analysis
• Relationship between variables	• Quantitative research	• ANOVA model, descriptive analysis and *t* test

## Results and Discussion

According to the methodological process described in this manuscript, Table 2 shows the profile (descriptive analysis) of the participants who answered this questionnaire. In this analysis, it is possible to note the differences and similarities between males and females, 89.97 and 10.03%, respectively, as well as the high proportion of repatriated and deported migrants (55.77% in total).

**TABLE 2:** Descriptive statistics.

	**Number of participants by type of NPOs**		**Total (percentage)**
		
	**Religious institutions**	**Civil NPOs**	
**Gender** • Male • Female	609 (39.67%) 144 (9.38%)	772 (50.3%) 10 (0.65%)	1381 (89.97%) 154 (10.03%)
**Migratory status** • Repatriation • Deportation • In transit/transmigrant/other	264 (17.20%) 283 (18.44%) 206 (13.42%)	83 (5.41%) 226 (14.72%) 473 (30.81%)	347 (22.61%) 509 (33.16%) 679 (44.24%)
Total	753 (49.05%)	782 (50.95%)	1535 (100%)

The version of the final questionnaire was an instrument consisting of different variables that measured volunteer commitment with items distributed randomly. Beneficiaries filled the questionnaire and this instrument with items was plotted in random order for self-administration by item sequentially. After the pre-test, the items were represented in 10 Likert terms because not many neutral responses were found.

The final questionnaire was administered in the different shelters by interviewers with physical presence. The instrument was sequentially applied in order to avoid biases, to validate the stability of the solutions obtained in each step, and to generalize the results beyond the sample ones obtained (Hair et al., 2010). Finally, the same instrument was applied in the two types of institutions, both religious and civil NPOs.

The statistical analysis started with the empirical identification of the variables. According to the reduced adaptation of the Zarzuela and Antón (2015) and Juaneda et al. (2017) scales, internal consistency (Cronbach’s alpha) was analyzed and the item-total correlation was evaluated. The overall reliability of the scale showed an adequate coefficient alpha and the analysis of the item-total correlation for all variables ranged from 0.734 to 0.865. Additionally, according to Hair et al. (2010) an exploratory factor analysis of principal components with Varimax rotation was applied on the data.

### Data Analysis and Discussion

In order to test the hypotheses H1 to H6, volunteer helping behavior was used for further analysis in terms of one item, which measured individually this construct. Regarding the hypotheses H1 to H3, the ANOVA model was applied and significant differences were found regarding the individual’s internalization of the value system of the organization (Table 3: see Sign. *p* values < 0.05). In terms of the interaction effect among variables on behavior, no statistically significant differences were found (Sign. *p* value > 0.05).

**TABLE 3:** Hypothesis regarding the effect of attitudinal features on volunteer helping behavior (H1–H3).

**Source of variation**	**Degrees of freedom**	**Root mean square**	***F***	**Sign**
Identification	1	0.485	0.211	0.646
Internalization	1	13.516	5.893	0.016
Attitude toward NPOs	1	2.900	1.264	0.262
Identification × internalization	1	1.190	0.519	0.472
Identification × attitude toward NPOs	1	7.018	3.060	0.081
Internalization × attitude toward NPOs	1	1.673	0.729	0.394
Identification × internalization × attitude toward NPOs	1	0.910	0.397	0.529

In this research, the results showed that volunteer helping behavior was influenced by the individual’s internalization of the value system of the organization, related to the attitudinal values (that is, hypotheses H1 to H3). Moreover, it was not allowed to appreciate a significant relationship on the helping behavior in terms of interaction.

Given the exploratory character of this manuscript, the results of this work pointed out that these individual opinions and perceptions of beneficiaries on volunteer commitment are the basis of helping behavior in NPOs. Our findings of this analysis confirmed that volunteers in NPOs recognize the value system of the organization; they are also more likely to facilitate their decisions on what their institutions offer to beneficiaries.

Additionally, when a volunteer reflects a helping behavior supported by internal values and goals linked to those adopted by the organization, internalization implies an active manner to serve one’s own interests. This argument suggests that there is an interest in facilitating integration under the idea that the shelter will continue as a place of lodging for migrants, and beneficiaries are likely to be satisfied (Juaneda et al., 2017; Silva, 2019).

By contrast to the literature review of the conceptual framework abovementioned in previous pages, the individual’s identification of the value system of the institution and individual’s attitude toward NPOs are important only for certain respondents in this work; that is, only H2 was accepted (see the results in Table 3).

Table 4 describes the statistical analysis of hypotheses H4 to H6 and the interaction effect among the normative attributes toward the NPO and social problems.

**TABLE 4:** Hypothesis related to the effect of normative features on volunteer helping behavior (H4–H6).

**Source of variation**	**Degrees of freedom**	**Root mean square**	***F***	**Sign**
Gratitude toward the NPO	1	1.438	0.650	0.420
Moral obligation toward the NPO	1	66.241	29.941	0.000
Attitude toward social problems	1	0.317	0.143	0.705
Gratitude × moral obligation	1	0.584	0.264	0.607
Gratitude × attitude toward social problems	1	0.298	0.135	0.714
Moral obligation × attitude toward social problems	1	0.791	0.358	0.550
Gratitude × moral obligation × attitude toward social problems	1	0.013	0.006	0.940

Similarly, in order to evaluate the contribution of normative aspects to the variance in volunteer helping behavior according to the beneficiaries’ perceptions, the results of this ANOVA model highlighted that these attributes have a direct effect in terms of the individual’s sense of moral obligation toward the NPO (Sign. *p* value < 0.05), but no significant effect of interaction was founded (Sign. *p* value > 0.05).

Results show that beneficiaries perceive that helping behaviors in shelters diminish the social, economic, and psychological costs providing opportunities to regularize their status. Furthermore, according to the academic literature on volunteer commitment, NPOs provide accommodation, food, and clothing but shelter organizations play an important role as communities of reception for beneficiaries, linking migrants to residents and providing emotional and spiritual support (Tekingündüz et al., 2017; Silva, 2019). In summary, hypotheses H4 to H6 were tested and only H5 was accepted.

Table 5 presents descriptive statistics for the variables of volunteer commitment. As a clear evidence, the detailed analysis reveals high scores in the “individual’s internalization of the value system of the organization” in religious institutions (value: 9.30) and “moral obligation” in civil NPOs (value: 9.77). Also, “attitude toward social problems” was one of the best valued items in both type of institutions.

**TABLE 5:** Descriptive statistics on volunteer helping behavior.

	**Religious institutions**	**Civil NPOs**	**Values**	
			**Min**	**Max**
**Attitudinal commitment**				
Identification	8.45	8.16	5	10
Internalization	9.30	8.18	5	10
Attitude toward NPOs	8.14	8.70	1	10
**Normative commitment**				
Gratitude	9.17	8.58	5	10
Moral obligation	9.77	8.18	7	10
Attitude toward social problems	8.63	8.88	5	10

The result of this analysis provided additional evidences and our findings suggest that statistical differences on most of the variables could be found. For evaluating the relevance of further information concerning volunteering, the effect of volunteer commitment was measured using the helping behavior as the dependent variable. Table 6 shows group-mean values; additionally, a *t* test analysis by type of institutions, that is religious versus civil NPOs, and gender of the respondent was conducted.

**TABLE 6:** *t* test, means (M), and standard deviation (SD) on volunteer helping behavior.

		**Type of NPOs**				**Gender**			
			
	**M/SD**	**Religious institutions**	**Civil NPOs**	***t***	**Sign.**	**Male**	**Female**	***t***	**Sign**
Helping behavior	6.41/1.83	7.64	5.22	13.774	0.000	6.54	5.24	5.085	0.000

The results suggest that statistical differences were found, with significantly lower values for civil NPOs than the religious ones. This result is according to the review of the literature (Ranganathan and Henley, 2008; Van Vuuren et al., 2008; Abdul, 2015; Ohana and Meyer, 2016; Metz et al., 2017; Tekingündüz et al., 2017; Ariza-Montes et al., 2018; Silva, 2019).

The findings reported in this study confirm that Centro Madre Assunta and Casa de los Migrantes were evaluated differently according to the information given (see Table 6). Similar evidences have been reported in several studies (Briggs et al., 2007; Ranganathan and Henley, 2008; Ohana and Meyer, 2016; INEGI, 2017; Metz et al., 2017; Silva, 2019). In terms of gender, our results evidenced that statistical differences between males and females were found (Ariza-Montes et al., 2015).

The main reason for this differentiated consideration is that previous studies have found that migrants intermingle in civil shelters, but in religious institutions, beneficiaries find ways to relate to each other, creating a singular environment to belong to any particular faith. In an extended perspective, in the religious shelters, managers see migrants as beneficiaries to engage in ministry in both a humanistic and religious sense.

In the San Diego–Tijuana area, religious NPOs include Catholic institutions, Protestant, as well as Baptist–Pentecostal churches playing a relevant role as communities of reception, linking migrants to long-term residents, providing emotional support and creating bonds among beneficiaries, volunteers, and shelter coordinators that persist after migrants’ departure (Brudney and Meijs, 2014; Ohana and Meyer, 2016; INEGI, 2017; Silva, 2019).

## Conclusion, Implications, Limitations, and Further Research

Relevant findings grounded in theoretical and empirical implications are required about the relevance of being alert to the feelings and needs and of volunteers in NPOs. Although many research aspects have to be considered in the volunteers’ decisions to cooperate with NPOs, this paper is focused on descriptions on their behaviors when they are participating in crisis actions such as cross-border conflicts.

There is some evidence that HR management can be improved through appropriate practices. Social interventions of NPOs have resulted in significant gains and improved volunteer commitment and helping behaviors worldwide, and as a result, these actions have increased satisfaction of beneficiaries, managers, and volunteers in shelters.

To sum up, this research has analyzed the effects of attitudinal and normative features of the volunteer commitment on helping behavior. Regarding the six hypotheses presented in this paper, the individual’s attitudes and normative-focused values are evidenced as relevant factors to determine the helping behavior of the volunteers in NPOs from the beneficiaries’ perspective. That is, the individual’s internalization with the value system of the NPO and the sense of moral obligation are key indicators for evaluating the volunteer helping behavior, showing a relevant signal for managers in HR management practices.

In terms of the main theoretical contributions and practical implications that emerge from this study, our results reveal that internalization and moral obligation significantly influence the volunteer helping behavior. Regarding the NPOs, affective and normative features are very relevant aspects to be taken into account for managers. These aspects are also best valued for beneficiaries; for this reason, current conditions pose a challenge for the NPOs in order to integrate repatriated or deported migrants deciding to stay or settle permanently in a city such as Tijuana. Each of these particularities for migrants implies a set of specific needs with respect to social services, employment, and medical attention.

From a theoretical approach, there are not many studies that describe the particular relationships described, here, mainly because of the unequal presence of cross-border conflicts around the world. Considering implications for practitioners, these findings are relevant for institutional representatives and managers in NPOs, taking into account not only the costs and benefits of implementing diverse HR practices but also aspects related to improve perceptions and experiences in NPOs when chiefs and managers are promoting their shelters. Thus, attitudinal features and a sense of moral obligation are aspects and factors whose combination facilitates to improve the level of service of the NPOs. Moreover, managers in NPOs should take into account the characteristics of beneficiaries when they are assisting migrants. Empirical results evidence that successful institutions have expanded their services to adjust to these new realities related to immigration, not only when formulating a plan to address the problem.

In this study, different combinations of attributes of volunteer commitment are considered; for this reason, it is possible that directors of the religious and civil establishments are oriented by social, utilitarian, and ethical criteria regarding the level of importance in attitudinal and normative concern when recruiting staff in NPOs.

Furthermore, it is interesting to consider also other types of personal services offered in NPOs such as those related to social, legal services, or assistance for employment, for example. In this work, a relevant aspect to be considered is that only migrants in face-to-face, that is, personal interviews and traditional shelters have participated in this research project. We did not test specific factors and variables such as ethnic extraction, religious beliefs, modes of operation in NPOs, or categories; for this reason, conclusions and implications should be only adapted in personal experiences promoting volunteering in NPOs. It will be relevant and interesting to further research related to environments with different profiles of beneficiaries (deported migrants, repatriated, transmigrants) or testing moderator and mediator effects.

To sum up, this work is particularly focused on a limited number of variables for each dimension of the volunteer commitment: attitudinal and normative-focused values. In essence, the main limitation of this research is both descriptive and exploratory in nature, particularly derived from the method used that does not predict the behavior itself. In this research, an important aspect to be considered is that we did not test specific variables predicting behaviors; in this sense, conclusions should be taken into account when the intention of conduct is not manifested in the action (conduct performed). Given that we include data from different shelters and institutions, further research considering moderating impacts with multi-group analysis will be of interest.

Finally, it is also evidenced that mass media and social networks provide a rapid, direct, and involved way to transmit any available information to beneficiaries; this consideration is a key factor for volunteering because they affect the way in which beneficiaries perceive the successful actions of NPOs. Additionally, further lines of research would highlight on the manner in which volunteers are deep acting and learning in their workplace; this is also of great value for managers in NPOs in order to improve their actions in the uncertain current environment.

## Data Availability Statement

The datasets generated for this study are available on request to the corresponding author.

## Ethics Statement

Ethical review and approval was not required for the study on human participants in accordance with the local legislation and institutional requirements. Written informed consent from the participant was not required to participate in this study in accordance with the national legislation and the institutional requirements.

## Author Contributions

All authors listed have made a substantial, direct, and intellectual contribution to this research. LC designed the study, wrote the manuscript, and analyzed the data. JV assisted in writing of the manuscript. MJ contributed by designing the study and collecting data, and reviewed the results and discussion. MR assisted in the writing and reviewed the entire manuscript.

## Conflict of Interest

The authors declare that the research was conducted in the absence of any commercial or financial relationships that could be construed as a potential conflict of interest.
