# Supplementary material for: Managing Cross-Border Conflicts Through Volunteer Commitment: A Comparative Study Between Religious and Non-profit Organizations in the San Diego–Tijuana Area
Source: Front Psychol. 2020 Jan 28;10:2978. doi: 10.3389/fpsyg.2019.02978 (PMC6997463; doi:10.3389/fpsyg.2019.02978)
Supplement: Supplementary file 1 [file Data_Sheet_1.PDF]

Annex: Questionnaire

Part I: ‘Volunteer helping behavior’.

Description of the item	From 1 = strongly disagree … to 10 = strongly agree							
The volunteer is willing to devote time and effort to volunteering work	1	2	3	…	…	…	…	10

Part II: ‘Attitudinal and normative-focused commitment’.

Description of the item	From 1 = strongly disagree … to 10 = strongly agree							
The volunteer is deeply committed to its organization	1	2	3	…	…	…	…	10
The volunteer is proud to belong to its organization	1	2	3	…	…	…	…	10
The organization is a good place to work	1	2	3	…	…	…	…	10
The volunteer is willing to socially participate in the organization	1	2	3	…	…	…	…	10
The volunteer conveys favorable information about the organization	1	2	3	…	…	…	…	10
The volunteer positively assess the goals that its organization has	1	2	3	…	…	…	…	10
Identification with the values promoted by the organization	1	2	3	…	…	…	…	10
The volunteer is concerned about the future of the organization	1	2	3	…	…	…	…	10
Feeling problems of the organization as its own	1	2	3	…	…	…	…	10
A great effort for this organization by the volunteer	1	2	3	…	…	…	…	10
Working in this NPO is better than in another one with similar activities.	1	2	3	…	…	…	…	10
The volunteer is very sensitive of the social problems in the border	1	2	3	…	…	…	…	10
NPOs are deeply committed to provide help for social problems	1	2	3	…	…	…	…	10
Solving social problems is more importance than economic growth	1	2	3	…	…	…	…	10
The volunteer receives more from the organization than he/she gives	1	2	3	…	…	…	…	10
The volunteer is indebted for what the organization has done for him/her	1	2	3	…	…	…	…	10
The volunteer is morally obliged to continue in its organization	1	2	3	…	…	…	…	10
The volunteer is loyal to its organization	1	2	3	…	…	…	…	10
The volunteer has a conscience to continue in the organization	1	2	3	…	…	…	…	10
Remaining is consistent with the way of thinking of the volunteer	1	2	3	…	…	…	…	10
The work done by NGOs is necessary to help the migrants	1	2	3	…	…	…	…	10
Everybody should be involved in some way or another with an NGO	1	2	3	…	…	…	…	10
There are no other more effective ways of helping than NGOs	1	2	3	…	…	…	…	10
Showing solidarity should begin with immigrants, the elderly, etc.	1	2	3	…	…	…	…	10
The volunteer is deeply concerned about social problems in the border	1	2	3	…	…	…	…	10
Possibility to do something by volunteer coupled with a wish to do it	1	2	3	…	…	…	…	10

Part III: 'Identification data of the respondent'.

1. **Gender**:
   - ☐ Male
   - ☐ Female
2. **Marital status**:
   - ☐ Single / Divorced / Widowed
   - ☐ Married
   - ☐ Other
   - ☐ No response
   - ☐ Centro Madre Assunta
3. **Level of education**:
   - ☐ None
   - ☐ Primary
   - ☐ Secondary
   - ☐ Tertiary
4. **Place of origin**: _________________
5. **Date of birth**: __________________
6. **Location / shelter**:
   - ☐ Centro Madre Assunta
   - ☐ Casa de los Migrantes
   - ☐ YMCA
   - ☐ Módulo Fronterizo Tijuana
7. **Migratory status**:
   - ☐ Repatriation
   - ☐ Deportation
   - ☐ In transit/transmigrant/other
